# The Action on Mucous Membranes of Silver Salts, with Especial Reference to Some of Their New Organic Forms

**Published:** 1903-08

**Authors:** Arthur G. Hobbs

**Affiliations:** Atlanta, Ga., Ophthalmic and Aural Surgeon to Presbyterian Hospital


### THE ACTION ON MUCOUS MEMBRANES OF SILVER SALTS, WITH ESPECIAL REFERENCE TO SOME OF THEIR NEW ORGANIC FORMS.*

BY ARTHUR G. HOBBS, M.D., ATLANTA, GA.,
Ophthalmic and Aural Surgeon to Presbyterian Hospital.

To discuss the therapeutic action of the silver salts from any point of view, the nitrate is the first that would naturally present itself to our minds, since it has been one of the staples in materia medica from time immemorial.

*Read before the Georgia State Medical Association, April 17, 1903.

Its potency is so great that ofttimes its misuse has resulted in much damage. In ignorant hands this inorganic form of silver is a dangerous remedy. Like all other things of potentiality, its misuse may result in as much disaster as its proper use may do good. Like fire, nitrate of silver should be applied with discretion—good in its place and in proper strengths—but equally as bad when used without thought.

Should I not say that it is, from a clinical standpoint, more like electricity, potential either for good or evil, according to the intelligence of him who applies it? Yet many of its disadvantages in some cases prove to be its advantages in others, and the reverse is equally true. The nitrate of silver can be used much stronger, with not only better results but with much less of its objectionable features, on squamous mucous membranes, such for example as we find on the deglutition part of the throat, than upon the columnar, and more especially upon the columnar-ciliated mucous membranes. Its irritative and caustic properties are especially objectionable to both the columnar and the columnar-ciliated form of epithelia.

We may in a general way remember that mucous membranes that are subjected to wear and tear are of the squamous variety, the columnar next and then the columnar-ciliated. The last especially resists less the contact or touch of anything foreign, and especially of anything that is irritating or caustic.

A much stronger solution of the inorganic salt can be applied, and advantageously so, to the lower pharynx and tonsils, than to the upper pharynx and nasal mucous membranes, and a still lesser strength must be applied to the Eustachian tube with its columnar-ciliated epithelium.

This rule applies to all other mucous membrane tracts and cavities. Hence one of the chief objections to our old standby—the nitrate of silver—is its irritating after-effects, and this applies whether we use it on the conjunctiva, in the urethra or on the nasal mucous membrane, with the exception only that mucous membranes of the squamous variety are always and under all circumstances benefited by it when the strength is properly considered. But when it is used on either a columnar or a columnar-ciliated epithelium in any considerable strength, it coagulates and destroys the outer layers and does not penetrate into the mucous membrane layer proper. It is upon this idea alone that all the new organic preparations of silver are or can be based.

Yet one more word about the inorganic form: In its greater strength it does just what we want it to do, under certain conditions. It cauterizes and destroys already broken-down tissues and stimulates a new and healthy growth beneath, but just here comes the questions: How strong shall it be? When and where shall we use it? What is the character of the membrane upon which we should use it?

Nitrate of silver is not yet relegated as a past number, and perhaps it never will be, however many substitutes it may have, since there are some forms of mucous membrane necroses in which it is an ideal applicant, not to mention the many other conditions in which it is most appropriate in its weaker forms.

It is somewhat analogous in its effects to quinine in its action in its different strengths. As quinine is tonic, antipyretic or antiphlogistic according to its dose, so is nitrate of silver stimulant, sedative and caustic, according to where it is applied and in what strengths. If applied too strong it may produce a stricture in the urethra, a stenosis in the nasal cavity, or a symblepharon of the conjunctiva.

And now we are brought face to face with some of the claims of the new organic forms of the salt after having placed our reliance for so many years upon this one that is inorganic.

Some of the recent proteid silver salts have in a measure, and under certain conditions, already taken the place of our standby, and justly so. They are better than it in some cases, because they are eliminated of its irritating and caustic properties.

Protargol, argyrol and albargin are the commercial names given to some of the most prominent of these organic salts of silver. The first, protargol, I believe, has been the most useful as a substitute of the nitrate in genito-urinary practice, but it can be so substituted, and with much advantage to the mucous membranes of all other tracts and cavities, especially where the lining is either columnar or columnar-ciliated, and when the subjective irritation is a consideration.

I shall not go into its genito-urinary adaptations, since I have had no experience with its use in that line, but suffice it to say that if we can judge by its voluminous literature, it would seem to have almost superseded the nitrate, just as argyrol is superseding the older salt in the ophthalmias.

Even the old and classical Crede method is rapidly giving way to the use of argyrol in the largest lying-in-hospitals, not only in this country but in Europe, mainly because of its non-irritant and non-caustic properties, but also because it penetrates deeper into the mucus and is more decidedly bacteriological in its effects. Albargin, which contains a much less percentage of silver than argyrol, is a gelatose silver, and hence seems to give up its base more gradually, more slowly, and to have deeper penetrating properties than some of the others, and for these reasons, in some cases, may produce a more desirable effect. While there are still other proteid organic compounds of silver, all of which are based upon the idea of substituting, or improving upon, the old inorganic form, I have not had enough experience with them to do them justice just now.

The object of this paper has simply been to call attention to the fact that we must recognize and test this new idea, that so many chemists are now working upon, viz.: that some ideal silver salt can be produced upon an organic or proteid basis that will eliminate the objectionable features of the nitrate. The literature on the use of these new organic silver salts has been voluminous during the past few years, and I must refer you to it in this short paper for the exact indications and strengths of each.

But I will herewith attach a full bibliography that you may have if you wish to pursue the subject as to the adaptation of each.

As I have already stated, it has been my object only to impress the fact that we must recognize this comparatively new idea of silver in its organic compounds and that we must finally decide which of them we shall use, when we shall use it, upon what mucous membrane and in what strength.

### SOME OF THE BIBLIOGRAPHY OF PROTARGOL, ALBARGIN AND ARGYROL.

Dr. Ludwig Weiss, New York, Jour. Am. Med. Assn., Jan. 24, 1903.
Dr. A. G. Hobbs, Atlanta, Ophthalmic Record.
Dr. D. A. Sinclair, New York, Phila. Med. Jour., Feb. 14, 1903.
Dr. E. A. Schumann, Philadelphia, Northwest Lancet, Feb. 15, 1903.
Dr. S. W. Bandler, New York, Med. Record, Mar. 14, 1903.
Dr. A. L. Wolbarts, New York, Jour. Cutaneous Dis., Dec., 1901.
Dr. J. M. Thomson, Boston, Med. Record, Aug. 16, 1902.
Dr. E. R. W. Frank, Berlin, Med. News, April 26, 1902.
Dr. J. Vanderpoel, New York, Med. Record, Feb. 22, 1902.
Dr. E. O. Bardwell, Emporium, Pa., Med. News, Sept. 21, 1901.
Dr. J. J. Kyle, Indianapolis, Amer. Jour. of Ophthal., July, 1901.
Dr. G. A. Davis, Summit Point, W. Va., Charlotte Med. Journal, April, 1902.
Dr. A. Alt, St. Louis, Amer. Jour. of Ophthal., Apr., 1901.
Drs. C. D. Westcott and B. Pussey, Ry. Surgeon, Oct. 30, 1900.
Dr. W. L. Burrage, Boston, Boston Med. and Surg. Jour., Feb., 1901.
Dr. F. J. Balch, Boston, Boston Med. and Surg. Jour., Feb., 1901.
Dr. A. G. Hobbs, Atlanta, Charlotte Med. Jour.
Dr. W. A. Wells, Washington, D. C., Boston Med. and Surg. Jour., July, 1901.
Dr. G. K. Swinburne, New York, Med. News, May 18, 1901.
Dr. J. Vanderpoel, New York, Med. News, May 18, 1901.
Dr. Jas. Pedersen, New York, Med. News, May 18, 1901.
Dr. W. A. Holden, New York, Med. News, May 18, 1901.
Dr. Jas. Pedersen, New York, Med. Times, April, 1901.

